# Universal Target Learning: An Efficient and Effective Technique for Semi-Naive Bayesian Learning

**DOI:** 10.3390/e21080729

**Published:** 2019-07-25

**Authors:** Siqi Gao, Hua Lou, Limin Wang, Yang Liu, Tiehu Fan

**Affiliations:** 1 College of Software, Jilin University, Changchun 130012, China; 2 Key Laboratory of Symbolic Computation and Knowledge Engineering of Ministry of Education, Jilin University, Changchun 130012, China; 3 Department of Software and Big Data, Changzhou College of Information Technology, Changzhou 213164, China; 4 College of Computer Science and Technology, Jilin University, Changchun 130012, China; 5 College of Instrumentation and Electrical Engineering, Jilin University, Changchun 130012, China

**Keywords:** information theory, universal target learning, Bayesian network classifier

## Abstract

To mitigate the negative effect of classification bias caused by overfitting, semi-naive Bayesian techniques seek to mine the implicit dependency relationships in unlabeled testing instances. By redefining some criteria from information theory, Target Learning (TL) proposes to build for each unlabeled testing instance P the Bayesian Network Classifier BNC${}_{P}$, which is independent and complementary to BNC${}_{T}$ learned from training data T. In this paper, we extend TL to Universal Target Learning (UTL) to identify redundant correlations between attribute values and maximize the bits encoded in the Bayesian network in terms of log likelihood. We take the *k*-dependence Bayesian classifier as an example to investigate the effect of UTL on BNC${}_{P}$ and BNC${}_{T}$. Our extensive experimental results on 40 UCI datasets show that UTL can help BNC improve the generalization performance.

## 1. Introduction

Supervised learning is a machine learning paradigm that has been successfully applied in many classification tasks [1,2]. Supervised learning has widespread deployment in applications including medical diagnosis [3,4,5], email filtering [6,7], and recommender systems [8,9,10]. The mission of supervised classification is to learn a classifier, such as neural network propagation and decision tree, from labeled training set T and then use it to assign class label *c* to some testing instance **x** = $\left\{x_{1},\cdots ,x_{n}\right\}$, where $x_{i}$ and *c* respectively denote the value of attribute $X_{i}$ and class variable *C*. Bayesian Network Classifiers (BNCs) [11] are such tools for indicating the probabilistic dependency relationships graphically and inferring under uncertainty conditions. They supply a framework to compute the joint probability, which can be written as the individual conditional probabilities of attributes given their parents, that is:

(1) $$P\left(c,x\right)=P\left(c|\pi_{c}\right)\prod_{i=1}^{n}P\left(x_{i}|\pi_{i}\right)$$

where $\pi_{i}$ and $\pi_{c}$ respectively denote the parents of attribute $X_{i}$ and that of class variable *C*.

Learning unrestricted BNCs is often time consuming and quickly becomes intractable as the number of attributes in a research domain grows. Moreover, inference in such unrestricted models has been shown to be NP-hard [12]. The success of Z-dependence Naive Bayes (NB) [13] has led to learning restricted BNCs or BNC${}_{T}$ from labeled training data T, e.g., one-dependence Tree Augmented Bayesian classifier (TAN) [14] and *k*-Dependence Bayesian classifier (KDB) [12]. Among them, KDB can generalize from one-dependence to an arbitrary *k*-dependence network structure and has received great attention from researchers in different domains. These BNCs attempt to extract from labeled training data the significant dependencies implicated, whereas overfitting may result in classification bias. For example, patients with similar symptoms sometimes may have diverse kinds of diseases, for example, VM (viral myocarditis) [15] is often diagnosed as influenza due to the low incidence rate.

Semi-supervised learning methods generally apply unlabeled data to either reprioritize or modify hypotheses learned from labeled data alone [16,17,18]. These methods efficiently combine the expressed classification information of the labeled data with the information concealed in the unlabeled data [19]. These algorithms generally assume that The general assumption of this class of algorithms is that data points in high density regions likely belong to the same class simultaneously as decision boundary exists in low density regions [20]. However, the information carried by one single unlabeled instance may be overwhelmed by mass training data, and a wrongly-assigned class label may result in “noise propagation”. To address this problem, we presented the Target Learning (TL) framework [21], in which an independent Bayesian model BNC${}_{P}$ learned from testing instance P can work jointly with BNC${}_{T}$ and effectively improve BNC${}_{T}$’s generalization performance with minimal additional computation. In this paper, we present an expanded presentation of TL, Universal Target Learning (UTL), through dynamically adjusting dependency relationships implicated in one single testing instance at classification time to explore the most appropriate network topology. Conditional entropy is introduced as the loss function to measure the bits encoded in BNC in terms of log likelihood.

The remainder of the paper is organized as follows: Section 2 reviews the state-of-the-art-related BNCs. Section 3 shows the theoretical justification of the UTL framework and describes the learning procedure of KDB within UTL. The extensive experimental studies on 40 datasets are revealed in Section 4. To finalize, the final section shows the conclusions and the future work.

## 2. Preliminaries

A pair with <G,Θ> can formalize a Bayesian Network (BN). G represents the structure containing nodes and arcs with a directed acyclic graph. Nodes symbolize the class or attribute variable, and arcs correspond to dependency relationships existing between the child nodes and parent nodes. Θ represents the parameter set, which includes the conditional probability distribution of each node in G, namely $P_{B}\left(c|\pi_{c}\right)$ or $P_{B}\left(x_{i}|\pi_{i}\right)$, where $\pi_{i}$ and $\pi_{c}$ respectively denote the parents of attribute $X_{i}$ and that of class variable *C* in structure G. Facts proved that it is an NP-hard problem to learn an optimal BN [22]. To deal with the sticky complexity, some learning of restricted network structures is under research [23]. Thus, the joint probability distribution is defined as:

(2) $$P_{B}\left(c,x\right)=P\left(c\right)\prod_{i=1}^{n}P_{B}\left(x_{i}|c,\pi_{i}\right).$$

Taking advantage of the underlying network topology of B and Equation (2), a BNC computes $P_{B}\left(c|x\right)$ by:

(3) $$P_{B}\left(c|x\right)=\frac{P_{B}\left(c,x\right)}{P_{B}\left(x\right)}=\frac{P_{B}\left(c,x\right)}{\sum_{c\in \Omega_{C}}P_{B}\left(c,x\right)}=\frac{P\left(c\right)\prod_{i=1}^{n}P_{B}\left(x_{i}|c,\pi_{i}\right)}{\sum_{c\in \Omega_{C}}P\left(c\right)\prod_{i=1}^{n}P_{B}\left(x_{i}|c,\pi_{i}\right)}.$$

**Figure 1:**
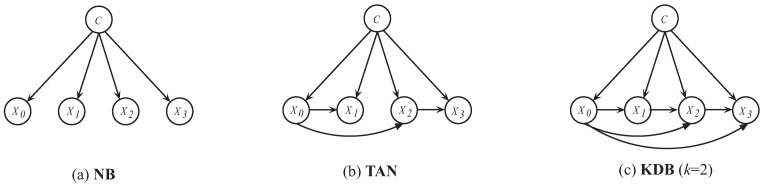
(**a**) NB; (**b**) Tree Augmented Bayesian classifier (TAN); (**c**) *k*-Dependence Bayesian classifier (KDB) (*k* = 2) with four attributes.

Among numerous restricted BNCs, NB is an extremely simple and remarkably effective approach with a zero-dependence structure (see Figure 1a) for classification [24,25]. It uses a simplifying assumption that given the class label, the attributes are independent of each other [26,27], i.e.,

(4) $$P_{\mathrm{NB}}\left(x|c\right)=\prod_{i=1}^{n}P\left(x_{i}|c\right).$$

However, in the real-world, NB’s attribute independence assumption is often violated and sometimes affects its classification performance. There has been generous prior work that explored methods to improve NB’s classification performance. Information theory, which was proposed by Shannon, has established a mathematical basis for the rapid development of BN. Mutual Information (MI) $I(X_{i};C)$ is the most commonly-used criterion to rank attributes for attribute sorting or filtering [28,29], and Conditional Mutual Information (CMI) $I(X_{i};X_{j}|C)$ is used to find conditional dependence between attribute pair $X_{i}$ and $X_{j}$ for identifying possible dependencies. $I(X_{i};C)$ and $I(X_{i};X_{j}|C)$ are defined as follows,

(5) $$\left\{\begin{array}{c} I\left(X_{i};C\right)=\sum_{x_{i}\in \Omega_{X_{i}}}\sum_{c\in \Omega_{C}}P\left(x_{i},c\right)log\frac{P(x_{i},c)}{P\left(x_{i}\right)P\left(c\right)}\\ I\left(X_{i};X_{j}|C\right)=\sum_{x_{i}\in \Omega_{X_{i}}}\sum_{x_{j}\in \Omega_{X_{j}}}\sum_{c\in \Omega_{C}}P\left(x_{i},x_{j},c\right)log\frac{P(x_{i},x_{j}|c)}{P\left(x_{i}|c\right)P\left(x_{j}|c\right)}. \end{array}\right.$$

The independence assumption may not hold for all attribute pairs, but may hold for some attribute pairs. Two categories of learning strategies have been proven effective based on NB. The first category aims at identifying the independency relationships to approximate NB’s independence assumption. Langley and Sage [27] proposed the wrapper-based Selective Bayes (SB) classifier, which carries out a greedy search through the space of attributes to accommodate redundant ones within the prediction process. Some methods relieve the violations of the attribute independence assumption through deleting strong related attributes (such as Backwards Sequential Elimination (BSE) [30] and Forward Sequential Selection (FSS) [31]). Some attribute weighting methods also achieve competitive performance. The earliest methods of weighted naive Bayes were proposed by Hilden and Bjerregaard [32], which used a single weight, then Ferreira [33] improved this by weighting each attribute value rather than each attribute. Hall [34] assigned the weight, which is in reverse ratio to the minimum depth at first tested in an uncorrected decision tree to each attribute. The other group introduced various categories to NB. Kwoh and Gillies [35] proposed a method that introduces one hidden variable to NB’s model as a child of the class label and as the parent of all predictor labels. Kohavi [36] described a hybrid approach that attempts to utilize the advantages of both decision trees and naive Bayes. Yang [37] proposed to fit NB’s conditional independence assumption by discretization.

The second category aims at relaxing the independence assumption by introducing the significant dependency relationships. TAN relaxes the independence assumption, as well as extends NB from the zero-dependence to the one-dependence maximum weighted spanning tree [14] (see Figure 1b). Based on this, Keogh and Pazzani [38] proposed to construct TAN by choosing the augmented arcs, which maximized the improvement of classification accuracy. ATAN [39] predicts by averaging each built TAN’s estimated class-membership probabilities. Weighted Averaged Tree-Augmented Naive Bayes (WATAN) [39] applies the aggregation weight by the mutual information between the class variable and root attribute. To represent more dependency relationships, an ensemble of one-dependence BNCs or high-dependence BNC is a feasible solution. RTAN [40] generates TAN, which describes the dependency relationships within a certain attribute sub-spaces. As a consequence, BaggingMultiTAN [40] trains these RTAN as component classifiers and is generated by the most votes. Averaged One-Dependence Estimators (AODE) [41] assumes that every attribute relies on the class and a shared attribute and only uses one-dependence estimators. To handle continuous variables, in every model, HAODE [42] considers a super-parent attribute’s discrete version, so that it can estimate the previous relationships by a univariate Gaussian distribution. As shown in Figure 1c (KDB with four attributes when *k* = 2), KDB can represent the arbitrary degree of dependency relationships and also achieve similar computational efficiency of NB [21]. Bouckaert proposed to average all of the possible network structures for the fixed value of *k* (containing lower orders) [43]. Rubio and Gámez presented a variant of KDB, which provided a hill-climbing algorithm to build a KDB incrementally [44].

To avoid high variance and classification bias caused by overfitting, how to mine the information existing in testing instance P is an interesting issue and has attracted more attention recently. Some algorithms try to combine P into training data T, which can help refine the network structure of classifier BNC${}_{T}$, which is learned from T only. The recursive Bayesian classifier [31] captures each predicted label provided by NB, and if misclassified, it induces a new NB from the cases that have the predicted label. A random oracle classifier [45] splits the labeled training data into two subsets using the random oracle and respectively trains two sub-classifiers. The testing instance then uses the random oracle to select one sub-classifier for classification. Other algorithms, though few, seek to explore the dependency relationships implicated in P only. Subsumption Resolution (SR) [46] identifies pairs of attribute-values in P, and if one is a generalization of the other, SR will delete the generalization. Target learning [21] extends P to a pseudo training set and then builds an independent BNC${}_{P}$ for it, which is complementary to BNC${}_{T}$ in nature.

## 3. UKDB: Universal Target Learning

### 3.1. Target Learning (TL)

Relaxing the independence assumption by adding augmented edges to NB is a feasible approach to refining NB and increasing the confidence level of the estimate of joint probability P(x,c). However, from Equation (5), we can see that, to compute MI or CMI, the (conditional) probability distributions needed are learned from labeled training dataset T only. Thus, as the structure complexity increases, the corresponding BNC may overfit the training data and underfit the unlabeled testing instance. This may lead to classification bias and high variance. To address the issue, we proposed the TL framework to build a specific BNC${}_{P}$ for any testing instance P at classification time to explore possible conditional dependencies that exist in P only. The BNC${}_{P}$ applies the same learning strategy as that of BNC${}_{T}$ learned from T. Thus, BNC${}_{P}$ and BNC${}_{T}$ are complementary to each other and can work jointly.

We take KDB as an example to illustrate the basic idea of TL. Given training dataset T, the learning procedure of KDB${}_{T}$ is shown in Algorithm 1.

**Algorithm 1:** The learning procedure of KDB${}_{T}$.

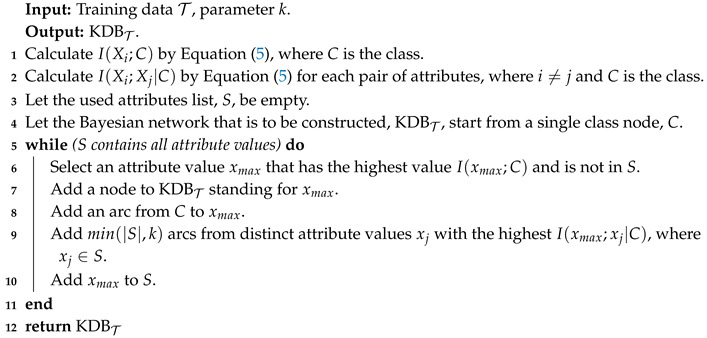



From the viewpoint of information theory, MI or $I(X_{i};C)$ can measure the mutual dependence between *C* and $X_{i}$. From Equation (5), we can see that $I(X_{i};C)$ is the expected value of mutual information over all possible values of *C* and $X_{i}$. Thus, although the dependency relationships between attributes may vary for different instances to a certain extent [21], the structure of traditional KDB cannot automatically fit diverse instances. To address the issue, for unlabeled testing instance $\left\{x_{1},\cdots ,x_{n}\right\}$, Local Mutual Information (LMI) and Conditional Local Mutual Information (CLMI) are introduced as follows to measure the dependency relationship between attribute values [21]:

(6) $$\left\{\begin{array}{c} \hat{I}\left(X_{i};C\right)=\sum_{c\in \Omega_{C}}P\left(x_{i},c\right)log\frac{P(x_{i},c)}{P\left(x_{i}\right)P\left(c\right)}\\ \hat{I}\left(X_{i};X_{j}|C\right)=\sum_{c\in \Omega_{C}}P\left(x_{i},x_{j},c\right)log\frac{P(x_{i},x_{j}|c)}{P\left(x_{i}|c\right)P\left(x_{j}|c\right)}. \end{array}\right.$$

Given training set *T*, KDB${}_{T}$ sorts attributes by comparing $I(X_{i};C)$ and chooses conditional dependency relationships by comparing $I(X_{i};X_{j}|C)$. In contrast, given testing instance $P=\{x_{1},x_{2},\cdots ,x_{n}\}$, KDB${}_{P}$ sorts attributes by comparing $\hat{I}\left(X_{i};C\right)$ and chooses conditional dependency relationships by comparing $\hat{I}\left(X_{i};X_{j}|C\right)$. The learning procedure of KDB${}_{P}$ is shown in Algorithm 2 as follows. 

**Algorithm 2:** The learning procedure of KDB${}_{P}$.

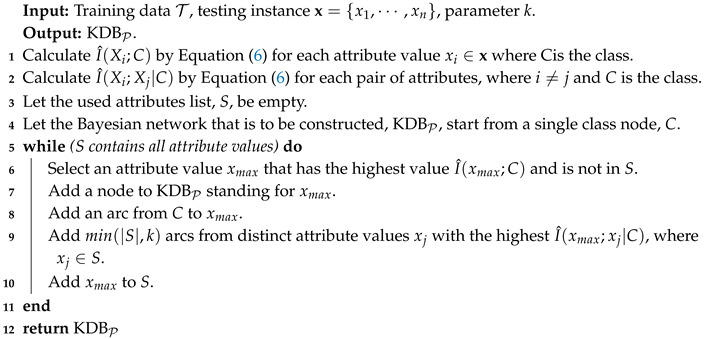



### 3.2. Universal Target Learning

Generally speaking, the aim of BNC learning is to find a network structure that can facilitate the shortest description of the original data. The length of this description considers the description of the BNC itself and the data applying the BNC [38]. Such a BNC represents a probability distribution $P_{B}\left(x\right)$ over the instance x appearing in the training data *T*.

Given training data *T* with *N* instances $T=\{d_{1},\cdots ,d_{N}\}$, the log likelihood of classifier B given *T* is defined as:

(7) $$LL\left(B|T\right)=\sum_{i=1}^{N}\log P_{B}\left(d_{i}\right),$$

which represents how many bits are required to describe T on account of the probability distribution $P_{B}$. The log likelihood has a statistical interpretation as well: the higher the log likelihood, the closer the classifier B is to model the probability distribution in T. The label of testing instance $U=\{x_{1},\cdots ,x_{n}\}$ may take any one of the |C| possible values of variable *C*. Thus, TL assumes that *U* is equivalent to a pseudo training set P that consists of |C| instances as follows,

(8) $$U=\left\{x_{1},\cdots ,x_{n}\right\}\Leftrightarrow P=\left\{\begin{array}{c} P_{1}=\left\{x_{1},\cdots ,x_{n},c_{1}\right\}\\ P_{2}=\left\{x_{1},\cdots ,x_{n},c_{2}\right\}\\ \cdots \\ P_{\left|C\right|}=\left\{x_{1},\cdots ,x_{n},c_{\left|C\right|}\right\} \end{array}\right.$$

Similar to the definition of LL(B|T), the log likelihood of classifier B given P is defined as:

(9) $$LL\left(B|P\right)=\sum_{i=1}^{\left|C\right|}\log P_{B}\left(P_{i}\right),$$

By applying different CMI criteria as shown in Equations (5) and (6), BNC${}_{P}$ and BNC${}_{T}$ provide two network structures to describe possible dependency relationships implicated in testing instances. These two CMI criteria cannot directly measure the bits that are needed to describe P based on $P_{B}$, whereas LL(B|P) can. From Equation (2),

(10) $$\begin{array}{cc} LL(B|P) & =\sum_{i=1}^{\left|C\right|}\log P_{B}\left(P_{i}\right)=\sum_{i=1}^{\left|C\right|}\log\left\{P\left(c_{i}\right)\prod_{j=1}^{n}P_{B}\left(x_{j}|c_{i},\pi_{j}\right)\right\}\\ & =\sum_{i=1}^{\left|C\right|}\log P\left(c_{i}\right)+\sum_{j=1}^{n}\sum_{i=1}^{\left|C\right|}\log P_{B}\left(x_{j}|c_{i},\pi_{j}\right)\\ & =\hat{H}\left(C\right)+\sum_{j=1}^{n}\hat{H}\left(X_{j}|C,\Pi_{j}\right) \end{array}$$

If there exist strong correlations between the values of parent attributes, we may choose to replace these correlations with meaningful dependency relationships. For example, let *Gender* and *Pregnant* be two attributes. If *Pregnant* = “yes”, it follows that *Gender* = “female”. Thus, *Gender* = “female” is a generalization of *Pregnant* = “yes” [46] and $P\left(Gender=‘‘female^{\prime \prime },Pregnant=‘‘yes^{\prime \prime }\right)=P\left(Pregnant=‘‘yes^{\prime \prime }\right)$. Given some other attribute values $\hat{x}=\left\{x_{1},\cdots ,x_{m}\right\}$, we can also have $P\left(Gender=‘‘female^{\prime \prime },Pregnant=‘‘yes^{\prime \prime },\hat{x}\right)=P\left(Pregnant=‘‘yes^{\prime \prime },\hat{x}\right)$. Correspondingly,

(11) $$\begin{array}{cc} P(x_{m+1}| & Gender=‘‘female^{\prime \prime },Pregnant=‘‘yes^{\prime \prime },\hat{x})=\frac{P(Gender=‘‘female^{\prime \prime },Pregnant=‘‘yes^{\prime \prime },\hat{x},x_{m+1})}{P(Gender=‘‘female^{\prime \prime },Pregnant=‘‘yes^{\prime \prime },\hat{x})}\\ & =\frac{P(Pregnant=‘‘yes^{\prime \prime },\hat{x},x_{m+1})}{P(Pregnant=‘‘yes^{\prime \prime },\hat{x})}=P\left(x_{m+1}|Pregnant=‘‘yes^{\prime \prime },\hat{x}\right) \end{array}$$

Obviously, for specific instances in which such correlations hold, the parent attribute *Gender* can not provide any extra information to $X_{m+1}$ and should be removed. To maximize LL(B|P), $X_{m+1}$ may select another attribute, e.g., $X_{p}$, as its parent to take the place of attribute *Gender*; thus, the dependency relationship between $X_{p}$ and $X_{m+1}$ that was neglected before can be added into the network structure. Many algorithms only explore improving the performance by removing redundant dependency relationships in the network structure, without considering to search for more meaningful dependency relationships. Because of the constraint of computational complexity that is closely related to structure complexity, each node in BNC can only take a limited number of attributes as parents. For example, KDB demands that at most *k* parents can be chosen for each node. Similarly, the proposed algorithm also follows this rule.

The second term in Equation (10), i.e., $\hat{H}\left(X_{j}|C,\Pi_{j}\right)$, is the log likelihood of conditional dependency relationships in B given P. To find proper dependency relationships implicated in each testing instance and maximize the estimate of LL(B|P), we need to maximize $\hat{H}\left(X_{j}|C,\Pi_{j}\right)$ for each attribute $X_{j}$ in turn. We argue that LL(B|P) provides a more intuitive and scalable measure for a proper evaluation. Based on the discussion presented above, in this paper, we propose to refine the network structures of BNC${}_{P}$ and BNC${}_{T}$ based on Universal Target Learning (UTL). In the following discussion, we take KDB as an example and apply UTL to KDB${}_{T}$ and KDB${}_{P}$ in similar ways, then we have UKDB${}_{T}$ and UKDB${}_{P}$ correspondingly. For testing instance P, UKDB${}_{T}$ or UKDB${}_{P}$ will recursively check all possible combinations of candidate parent attributes and attempt to find $\Pi_{j}$, which corresponds to the maximum of $\hat{H}\left(X_{j}|C,\Pi_{j}\right)$, that is $\Pi_{j}$ may contain less than min{i−1,k} attributes. By minimizing $\hat{H}\left(X_{j}|C,\Pi_{j}\right)$ for each attribute $X_{j}$, UKDB${}_{T}$ and UKDB${}_{P}$ are supposed to be able to seek more proper dependency relationships implicated in specific testing instance P and that may help to maximize the estimate of LL(B|P). For example, suppose that the attribute order of KDB${}_{T}$ is $\left\{X_{0},X_{1},X_{2},X_{3}\right\}$ and k=2, then for attribute $X_{2}$, its candidate parents are $\left\{X_{0},X_{1}\right\}$. Given testing instance P, we will compare and find $\Pi_{2}$ where $\hat{H}\left(X_{2}|C,\Pi_{2}\right)=\max\left\{\hat{H}\left(X_{2}|C,X_{0}\right),\hat{H}\left(X_{2}|C,X_{1}\right),\hat{H}\left(X_{2}|C,X_{0},X_{1}\right)\right\}$, and $\Pi_{2}\subset \left\{X_{0},X_{1},\left(X_{0},X_{1}\right)\right\}$. Thus, UKDB${}_{T}$ dynamically adjusts dependency relationships for different testing instances at classification time. Similarly, UKDB${}_{P}$ applies the same learning strategy to refine the network structure of KDB${}_{P}$.

Given *n* attributes, we can have n! possible attribute orders, and among them, the orders respectively determined by $I(X_{i};C)$ and $\hat{I}\left(X_{i};C\right)$ have been proven to be feasible and effective. Thus, for attribute $X_{i}$, its parents can be selected from two sets of candidates. The final classifier is also an ensemble of UKDB${}_{T}$ and UKDB${}_{P}$. UTL retains the characteristic of target learning, that is UKDB${}_{T}$ and UKDB${}_{P}$ are complementary, and they can work jointly to make the final prediction. The learning procedures of UKDB${}_{T}$ and UKDB${}_{P}$, which are respectively shown in Algorithms 3 and 4 as follows, are almost the same, except the pre-determined attribute orders.

In contrast to TL, UTL can help BNC${}_{P}$ and BNC${}_{T}$ encode the most possible dependency relationships implicated in one single testing instance. The linear combiner is appropriate to be used for models that output real-valued numbers, so it is applicable for BNC. For testing instance x, the ensemble probability estimate for UKDB${}_{T}$ and UKDB${}_{P}$ is,

(12) $$\hat{P}\left(y|x\right)=\alpha .P\left(y|x,UKDB_{T}\right)+\beta .P\left(y|x,{\mathrm{UKDB}}_{P}\right)$$

For different instances, the weights, α and β, may differ greatly, and there is no effective way to address issue. Thus, in fact, we simply use the uniformly- rather than non-uniformly-weighted average of the probability estimates. That is, we set α=β=0.5 for Equation (12).

**Algorithm 3:** UKDB${}_{T}$.

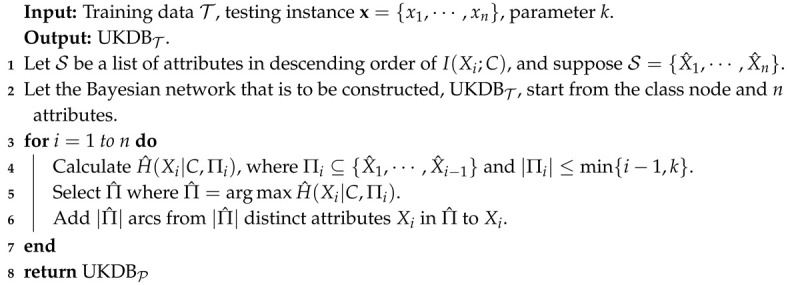



**Algorithm 4:** UKDB${}_{P}$.

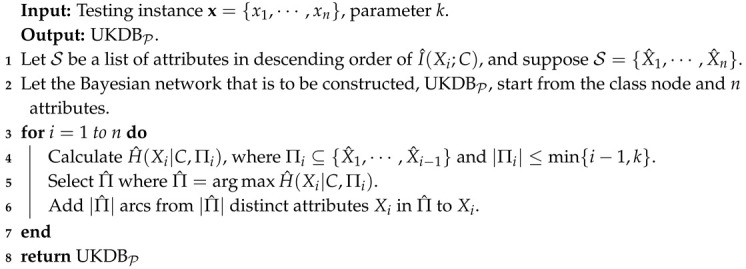



## 4. Results and Discussion

All algorithms for the experimental study ran on a C++ system (GCC 5.4.0). For KDB and its variations, as *k* increased, the time complexity and the structure complexity always increased exponentially. The *k* with larger values may contribute to promoting the classification accuracy in contrast to the smaller value of *k*. There are some requirements on *k* due to the constraint of currently available hardware resources. When k=3, UKDB’s experimental results on some large-scale datasets can not be tested due to the amount of CPU available. Thus, we only chose to select k=1 and k=2 in the following experimental study. To demonstrate the effectiveness of the UTL framework, the following algorithms (including three single-structure BNCs and an ensemble BNC) will be compared with ours,

- NB, the standard Naive Bayes.
- TAN, Tree-Augmented Naive Bayes.
- $K_{1}\mathrm{DB}$, *k*-dependence Bayesian classifier with *k* = 1.
- $K_{2}\mathrm{DB}$, *k*-dependence Bayesian classifier with *k* = 2.
- AODE, Averaged One-Dependence Estimators.
- WATAN, the Weighted Averaged Tree-Augmented Naive Bayes.
- TAN^*e*^, an ensemble Tree-Augmented Naive Bayes applying target learning.
- ${\mathrm{UK}}_{1}\mathrm{DB}$, *k*-dependence Bayesian classifier with *k* = 1 in the framework of UTL.
- ${\mathrm{UK}}_{2}\mathrm{DB}$, *k*-dependence Bayesian classifier with *k* = 2 in the framework of UTL.

We randomly selected 40 datasets from the UCI machine learning repository [47] for our experimental study. The datasets were divided into three categories, i.e., large datasets with the number of instances >5000, medium datasets with the number of instances >1000 and <5000, and small datasets with the number of instances <1000. The above datasets are described in Table 1 in detail, including the number of instances, attributes, and classes. All the datasets are ordered in ascending order of dataset size. The number of attributes ranged widely from 4–56, convenient for evaluating the effectiveness of the UTL framework to mine dependency relationships between attributes. Meanwhile, we can examine the classification performance with various sizes from 24 instances to 5,749,132 instances. Missing values were replaced with distinct values. We used Minimum Description Length (MDL) discretization [48] to discretize the numeric attributes.

To validate the effectiveness of UTL, the proposed UKDB are contrasted with three single-structure BNCs (NB, TAN, and KDB), as well as three ensemble BNCs (AODE, WATAN, TAN^*e*^) in terms of zero-one loss, RMSE, and $F_{1}$-score in Section 4.1. Then, we introduce the criteria, goal difference, and relative zero-one loss ratio, to measure the classification performance of UKDB while dealing with different quantities of training data and different numbers of attributes in Section 4.2 and Section 4.3, respectively. In Section 4.4, we compare the time cost for training and classifying. At last, we conduct the global comparison in Section 4.5.

### 4.1. Comparison of Zero-One Loss, RMSE, and $F_{1}$-Score

#### 4.1.1. Zero-One Loss

The experiments were tested by applying 10 rounds of 10-fold cross-validation. We used Win/Draw/Loss (W/D/L) to clarify the experimental results. To compare the classification accuracy, Table A1 in Appendix A reports the average zero-one loss for each algorithm on different datasets. The corresponding W/D/L records are summarized in Table 2.

As shown in Table 2, for the single-structure classifier, ${\mathrm{UK}}_{1}\mathrm{DB}$ performed significantly better than NB and TAN. Most importantly, ${\mathrm{UK}}_{1}\mathrm{DB}$ achieved significant advantage over $K_{1}\mathrm{DB}$ in terms of zero-one loss with 21 wins and only seven losses, providing convincing evidence for the validity of the proposed algorithm. For large datasets, the advantage was even stronger. Simultaneously, ${\mathrm{UK}}_{2}\mathrm{DB}$ achieved significant advantage over $K_{2}\mathrm{DB}$ with a W/D/L of 28/8/4. That is, $K_{2}\mathrm{DB}$ only achieved better results of zero-one loss over ${\mathrm{UK}}_{2}\mathrm{DB}$ on four datasets (contact-lenses, lung-cancer, sign, nursery); thus, ${\mathrm{UK}}_{2}\mathrm{DB}$ seldom performed worse than KDB. In contrast, ${\mathrm{UK}}_{2}\mathrm{DB}$ performed better than $K_{2}\mathrm{DB}$ more often on many datasets, such as car, poker-hand, primary-tumor, waveform-5000. When compared with the ensemble algorithms, ${\mathrm{UK}}_{1}\mathrm{DB}$ and ${\mathrm{UK}}_{2}\mathrm{DB}$ still enjoyed an advantage over AODE, WATAN, and TAN^*e*^. Moreover, the comparison results of ${\mathrm{UK}}_{2}\mathrm{DB}$ with AODE and WATAN were almost significant (24 wins and only three losses, 24 wins and only two losses, respectively). Based on the discussion above, we argue that UTL is an effective approach to refining BNC.

#### 4.1.2. RMSE

**Figure 2:**
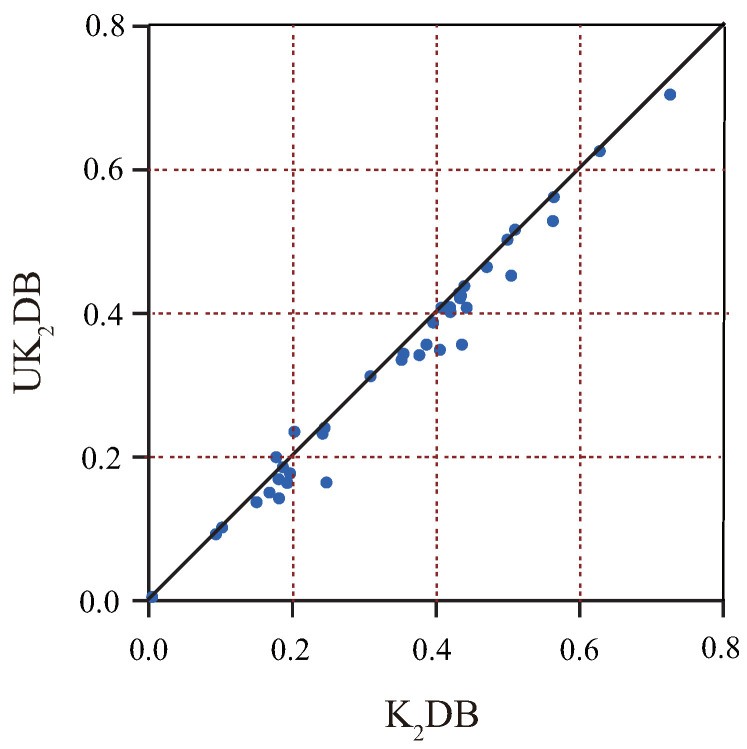
The scatter plot of ${\mathrm{UK}}_{2}\mathrm{DB}$ and $K_{2}\mathrm{DB}$ in terms of RMSE.

The Root Mean Squared Error (RMSE) is used to measure the deviation between the observed value and the true value [49]. Table A2 in Appendix A reports the RMSE results for each algorithm on different datasets. The corresponding W/D/L records are summarized in Table 3. The scatter plot between ${\mathrm{UK}}_{2}\mathrm{DB}$ and $K_{2}\mathrm{DB}$ in terms of RMSE is shown in Figure 2. The *X*-axis shows the RMSE results of $K_{2}\mathrm{DB}$, and the *Y*-axis shows the RMSE results of ${\mathrm{UK}}_{2}\mathrm{DB}$. We can observe that there are generous datasets under the diagonal line, such as labor-negotiations, lymphography and poker-hand, which shows that ${\mathrm{UK}}_{2}\mathrm{DB}$ has some advantages over $K_{2}\mathrm{DB}$. Simultaneously, except credit-a and nursery, the other datasets approach close to the diagonal line, which means ${\mathrm{UK}}_{2}\mathrm{DB}$ rarely performed worse than $K_{2}\mathrm{DB}$. For many datasets, UTL substantially helped reduce the classification error of $K_{2}\mathrm{DB}$, for example the reduction from 0.4362 to 0.3571 on dataset lymphography. As shown in Table 3, for the single-structure classifiers, ${\mathrm{UK}}_{1}\mathrm{DB}$ performed significantly better than NB and TAN. Moreover, ${\mathrm{UK}}_{1}\mathrm{DB}$ achieved significant advantages over $K_{1}\mathrm{DB}$ with 10 wins and four losses and ${\mathrm{UK}}_{2}\mathrm{DB}$ over $K_{2}\mathrm{DB}$ with 14 wins and the losses, which provides convincing evidence for the validity of the proposed framework. When compared with the ensemble group, ${\mathrm{UK}}_{1}\mathrm{DB}$ and ${\mathrm{UK}}_{2}\mathrm{DB}$ still had a significant advantage. ${\mathrm{UK}}_{1}\mathrm{DB}$ and ${\mathrm{UK}}_{2}\mathrm{DB}$ had obvious advantage with W/D/L of 10/24/6 and 24/13/3 when compared with AODE. ${\mathrm{UK}}_{2}\mathrm{DB}$ also achieved relatively significant advantage when coming to WATAN and TAN^*e*^ (14 wins and only two losses, 15 wins and only three losses). ${\mathrm{UK}}_{2}\mathrm{DB}$ reduced RMSE more substantially. UKDB not only performed better than single-structure classifiers, but also was shown as an effective ensemble model when compared with AODE in terms of RMSE.

#### 4.1.3. $F_{1}$-Score

Generally speaking, zero-one loss can roughly measure the classification performance of BNC, but it cannot evaluate whether the BNC can work consistently while dealing with different parts of imbalanced data. In contrast, precision gives the ratio of the true classification in all test data predicted to be true, and recall gives the ratio of the true classification in all test data actually to be true [50]. Precision and recall sometimes have contradictory situations; therefore, we employed the $F_{1}$-score, the harmonic average of the precision and recall, to measure the performance of our algorithm. In order to apply the multiclass classification problem, we employed the confusion matrix to measure the $F_{1}$-score. Suppose that there exists a dataset to be classified with the classes $\left\{C_{1},C_{2},\cdots ,C_{m}\right\}$. The confusion matrix as follows shows the classification results:

$$\left[\begin{array}{ccc} N_{11} & \cdots & N_{1m}\\ \vdots & \ddots & \vdots \\ N_{m1} & \cdots \; & N_{mm} \end{array}\right]$$

Each entry $N_{ii}$ of the matrix presents the number of instances, whose true class is $C_{i}$ that are actually assigned to $C_{i}$ (where 1≤i≤m). Each entry $N_{ij}$ presents the number of instances, whose true class is $C_{i}$, but nevertheless are actually assigned to $C_{j}$ (where i≠j and 1≤i,j≤m). Given the confusion matrix, precision, recall, and $F_{1}$-score are computed as follows:

(13) $$Precision_{i}=\frac{N_{ii}}{\sum_{j=1}^{m}N_{ji}}$$

(14) $$Recall_{i}=\frac{N_{ii}}{\sum_{j=1}^{m}N_{ij}}$$

(15) $$F_{1}-score_{i}=2\cdot \frac{Precision_{i}\cdot Recall_{i}}{Precision_{i}+Recall_{i}}$$

(16) $$F_{1}-score=\sum_{i=1}^{m}\frac{F_{1}-score_{i}}{m}$$

Table A3 in Appendix A reports the $F_{1}$-score for each algorithm on different datasets. Table 4 summarizes the W/D/L of the $F_{1}$-score. Several points in this table are worth discussing:

As shown in Table 4, for the single-structure classifiers, ${\mathrm{UK}}_{1}\mathrm{DB}$ performed significantly better than NB and TAN. When compared with the ensembles, ${\mathrm{UK}}_{1}\mathrm{DB}$ and ${\mathrm{UK}}_{2}\mathrm{DB}$ still had a slight advantage over AODE and achieved significant advantages over WATAN and TAN${}^{e}$. Most importantly, ${\mathrm{UK}}_{1}\mathrm{DB}$ performed better than $K_{1}\mathrm{DB}$ and ${\mathrm{UK}}_{2}\mathrm{DB}$ better than $K_{2}\mathrm{DB}$, although the advantage was not significant, which provides solid evidence for the effectiveness of UTL.

### 4.2. Goal Difference

To further compare the performance of UKDB with other mentioned algorithms in terms of data size, the Goal Difference (GD) [51,52] was introduced. Suppose for two classifiers *A*, *B*, we compute the value of GD as follows:

(17) GD(A;B|T)=|win|−|loss|.

where T represents the datasets for comparison and |win| and |loss| are respectively the numbers of datasets on which the classification performance of *A* is better or worse than that of *B*.

**Figure 3:**
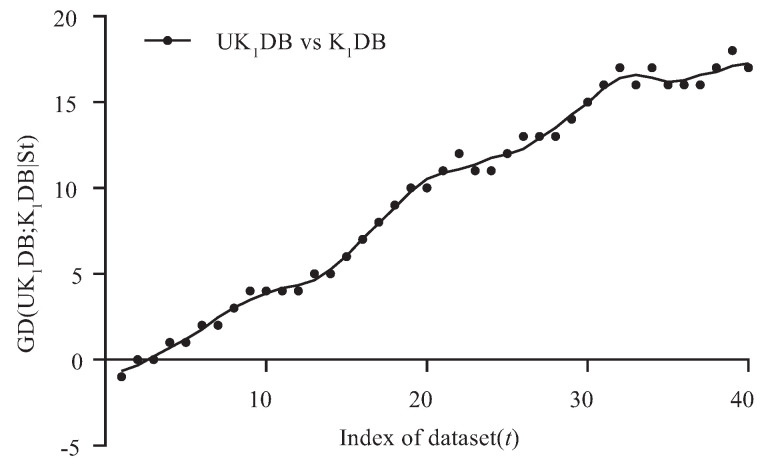
Goal Difference (GD(${\mathrm{UK}}_{1}\mathrm{DB}$; $K_{1}\mathrm{DB}$|T)) in terms of zero-one loss.

**Figure 4:**
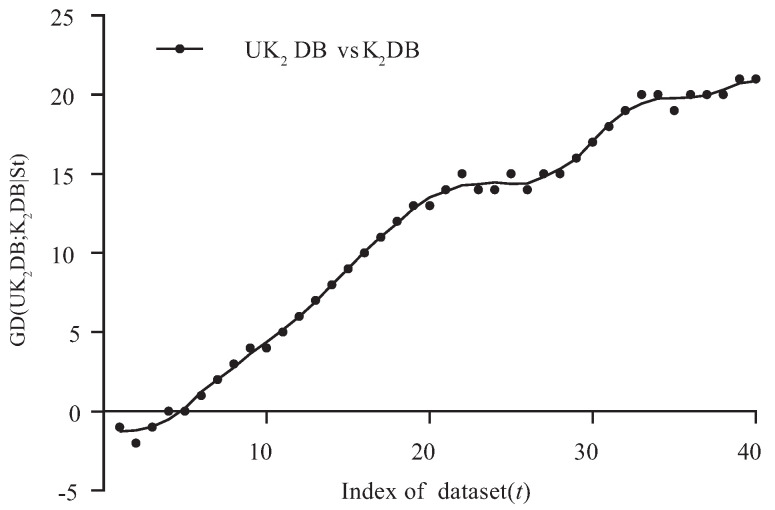
GD(${\mathrm{UK}}_{2}\mathrm{DB}$; $K_{2}\mathrm{DB}$|T) in terms of zero-one loss.

Figure 3 and Figure 4 respectively show the fitting curve of GD(${\mathrm{UK}}_{1}\mathrm{DB}$; $K_{1}\mathrm{DB}$|$S_{t}$) and GD(${\mathrm{UK}}_{2}\mathrm{DB}$; $K_{2}\mathrm{DB}$|$S_{t}$) in terms of the zero-one loss. The X-axis represents the indexes of datasets described in Table 1 (referred to as *t*), and the Y-axis respectively represents the values of GD(${\mathrm{UK}}_{1}\mathrm{DB}$; $K_{1}\mathrm{DB}$|$S_{t}$) and GD(${\mathrm{UK}}_{2}\mathrm{DB}$; $K_{2}\mathrm{DB}$|$S_{t}$), where $S_{t}$ denotes the collection of datasets, i.e., $S_{t}=\left\{D_{m}|m\leq t\right\}$ and $D_{m}$ is the dataset with index *m*.

From Figure 3, we can see that ${\mathrm{UK}}_{1}\mathrm{DB}$ achieved significant advantage over $K_{1}\mathrm{DB}$, and only on a few large datasets (nursery, seer-mdl, adult), the advantage was not obvious. Similarly, from Figure 4, we can see that there was an obvious positive correlation between the values of GD(${\mathrm{UK}}_{2}\mathrm{DB}$; $K_{2}\mathrm{DB}$|$S_{t}$) and the dataset size. The advantage of ${\mathrm{UK}}_{2}\mathrm{DB}$ over $K_{2}\mathrm{DB}$ was much more obvious than that of ${\mathrm{UK}}_{1}\mathrm{DB}$ over $K_{1}\mathrm{DB}$ on small and medium datasets. This superior performance is owed to the ensemble learning mechanism of UTL. UTL played a very important role in discovering proper dependency relationships that exist in testing instances. Since UTL replaces redundant dependency relationships with more meaningful ones, we can infer that UKDB retains the advantages of KDB, i.e., the ability to represent an arbitrary degree of dependence and to fit training data. This demonstrates the feasibility of applying UTL to search for proper dependency relationships. When dealing with large datasets, overfitting may lead to high variance and classification bias; thus, the advantage of UKDB over KDB was not obvious when k=1 or k=2.

**Figure 5:**
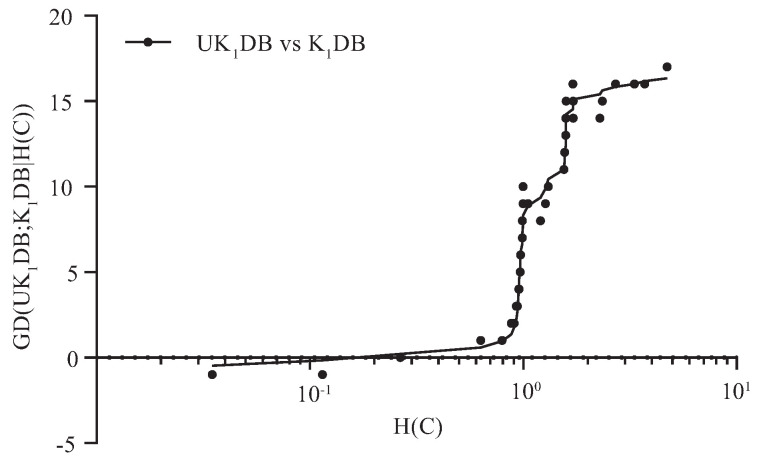
GD(${\mathrm{UK}}_{1}\mathrm{DB}$; $K_{1}\mathrm{DB}$|H(C)) in terms of zero-one loss.

**Figure 6:**
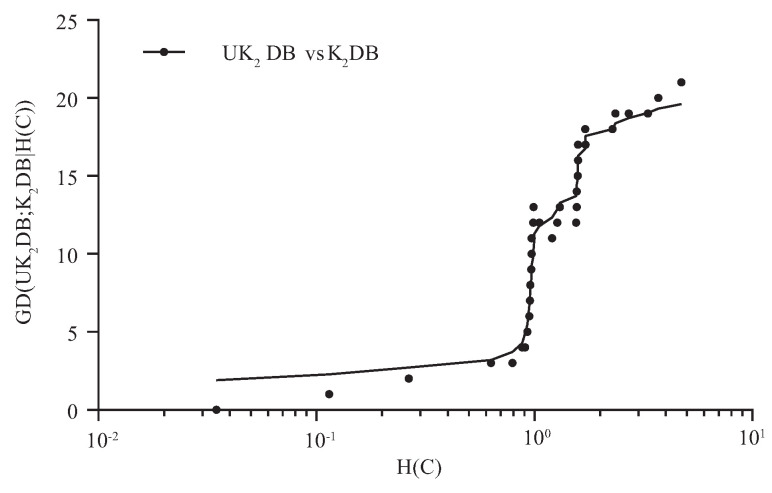
GD(${\mathrm{UK}}_{2}\mathrm{DB}$; $K_{2}\mathrm{DB}$|H(C)) in terms of zero-one loss.

For imbalanced datasets, the number of instances with different class labels will vary greatly, and that may lead to the estimate bias of the conditional probability. In this paper, the entropy function of class variable *C*, i.e., H(C), is introduced to measure the extent to which the datasets are imbalanced. UTL refines the network structure of BNC${}_{T}$ and BNC${}_{P}$ according to the attribute values rather than the class label of testing instance *U*. The negative effect caused by the imbalanced distribution of *C* will be mitigated to a certain extent. From Figure 5 and Figure 6, we can see that the advantage of UKDB over KDB becomes more and more significant as H(C)>0.8. Thus, these datasets with H(C)>0.8 are supposed to be relatively imbalanced and highlighted in Table A1, Table A2, and Table A3. Table 5 reports the corresponding H(C) values of these 40 datasets.

### 4.3. Relative Zero-One Loss Ratio

The criterion relative zero-one loss ratio can measure the extent of which classifier $A_{1}$ performs relatively better or worse than $A_{2}$ on different datasets. For instance, on dataset $D_{1}$, the zero-one losses of classifier $A_{1}$ and $A_{2}$ were respectively 55% and 50%; whereas on dataset $D_{2}$, the zero-one losses of classifier $A_{1}$ and $A_{2}$ were respectively 0% and 5%. Although the zero-one loss difference were always 5% for both cases, $A_{1}$ performed relatively better on dataset $D_{2}$ than $A_{2}$ on dataset $D_{1}$. Given two classifiers *A*, *B*, the relative zero-one loss ratio, referred to as $R_{Z}\left(\cdot \right)$, is defined as follows:

(18) $$R_{Z}\left(A|B\right)=1-\frac{Z_{A}}{Z_{B}}.$$

where $Z_{A(orB)}$ denotes the value of the zero-one loss of classifier A(orB) on a specific dataset. The higher the value of $R_{Z}\left(A|B\right)$, the better the performance of classifier *A* relative to classifier *B*.

**Figure 7:**
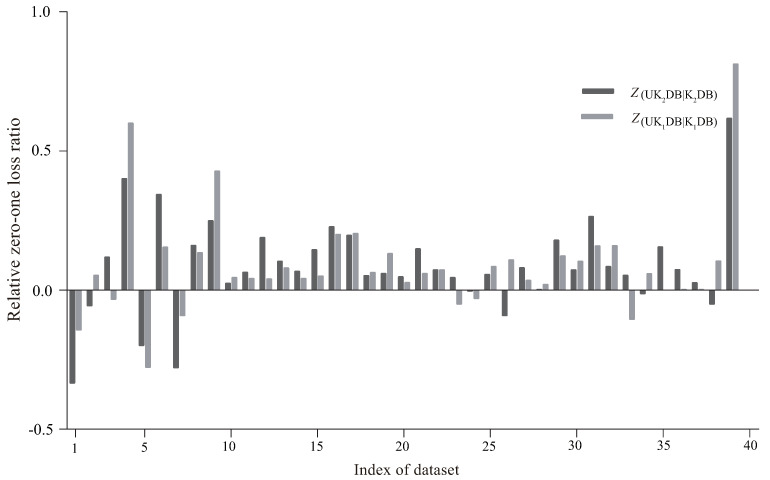
The comparison results of the relative zero-one loss ratio between UKDB and KDB when k=1 and k=2.

Figure 7 presents the comparison results of $R_{Z}\left(\cdot \right)$ of ${\mathrm{UK}}_{2}\mathrm{DB}$ and $K_{2}\mathrm{DB}$, ${\mathrm{UK}}_{1}\mathrm{DB}$, and $K_{1}\mathrm{DB}$. The X-axis represents the index of the dataset, and the Y-axis shows the value of $R_{Z}\left(\cdot \right)$. As we can observe intuitively, on most datasets, the values of $R_{Z\left({\mathrm{UK}}_{2}\mathrm{DB}|K_{2}\mathrm{DB}\right)}$ and $R_{Z\left({\mathrm{UK}}_{1}\mathrm{DB}|K_{1}\mathrm{DB}\right)}$ were positive, which demonstrates that UKDB achieved significant advantages over KDB no matter k=1 or k=2. Generally, in many cases, the difference between $R_{Z\left({\mathrm{UK}}_{2}\mathrm{DB}|K_{2}\mathrm{DB}\right)}$ and $R_{Z\left({\mathrm{UK}}_{1}\mathrm{DB}|K_{1}\mathrm{DB}\right)}$ was not obvious; thus, the working mechanism of UTL makes it insensitive to the structure complexity. For the first 10 datasets, the effectiveness of UTL was less significant. UK${}_{1}$DB beat K${}_{1}$DB on six datasets and lost on four, and UK${}_{2}$DB performed similarly. From Table 1, among these datasets on which UTL performed poorer, contact-lenses (No. 1), echocardiogram (No. 5), and iris (No. 7) had a small number of attributes, i.e., respectively 4, 6, and 4 attributes. A small dataset may lead to low confidence estimate of the probability distribution and then low-confidence estimate of $\hat{H}\left(X_{j}|C,\Pi_{j}\right)$. A small number of attributes makes it more difficult for UTL to adjust the dependency relationships dynamically. However, as the size of datasets increased, UKDB generally achieved more significant advantages over KDB. For the last 30 datasets, UTL only performed poorer on a few datasets, e.g., hypothyroid (No. 25), and among theses datasets, ${\mathrm{UK}}_{2}\mathrm{DB}$ worked much better than ${\mathrm{UK}}_{1}\mathrm{DB}$. From the above discussion, we can come to the conclusion that the UTL framework was effective at identifying significant conditional dependencies implicated in testing instance, whereas enough data for assuring high-confidence probability estimate was a necessary prerequisite.

### 4.4. Training and Classification Time

**Figure 8:**
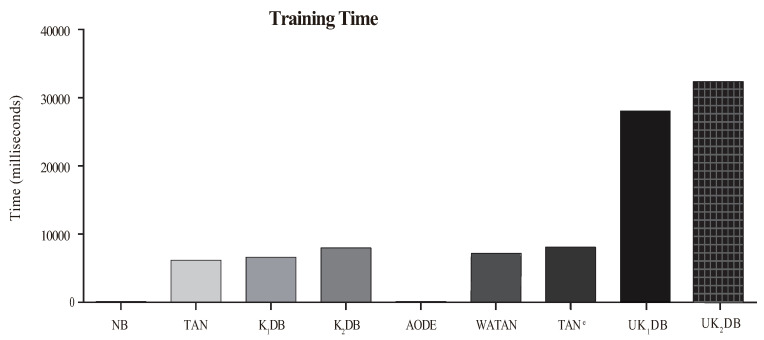
Training time of NB, TAN, $K_{1}\mathrm{DB}$, $K_{2}\mathrm{DB}$, AODE, WATAN, TAN^*e*^, ${\mathrm{UK}}_{1}\mathrm{DB}$, and ${\mathrm{UK}}_{2}\mathrm{DB}$.

**Figure 9:**
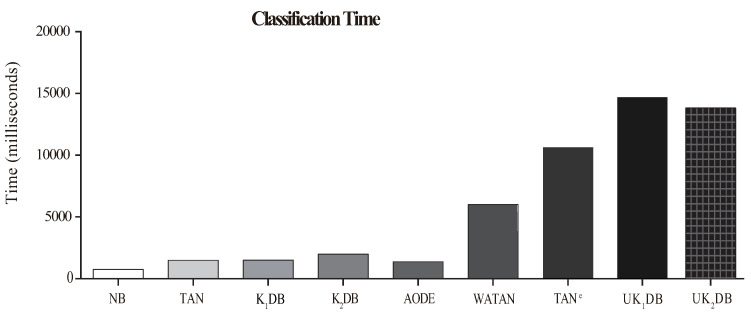
Classification time of NB, TAN, $K_{1}\mathrm{DB}$, $K_{2}\mathrm{DB}$, AODE, WATAN, TAN^*e*^, ${\mathrm{UK}}_{1}\mathrm{DB}$, and ${\mathrm{UK}}_{2}\mathrm{DB}$.

The comparison results of time for training and classifying are respectively displayed in Figure 8 and Figure 9. Each bar shows the sum time of 40 datasets.

From Figure 8, we can observe that our proposed algorithms ${\mathrm{UK}}_{1}\mathrm{DB}$ and ${\mathrm{UK}}_{2}\mathrm{DB}$ substantially needed more training time than the rest of the classifiers considered, i.e., NB, TAN, $K_{1}\mathrm{DB}$, $K_{2}\mathrm{DB}$, AODE, WATAN, and TAN^*e*^. ${\mathrm{UK}}_{2}\mathrm{DB}$ spent slightly more training time than ${\mathrm{UK}}_{1}\mathrm{DB}$ on account of more dependency relationships existing in ${\mathrm{UK}}_{2}\mathrm{DB}$. On the other hand, as shown in Figure 9, due to the ensemble learning strategy of UTL, NB, TAN, AODE, $K_{1}\mathrm{DB}$, and $K_{2}\mathrm{DB}$ consumed less classification time than UKDB when *k* = 1 or *k* = 2. This was due to the fact that during the learning process, UTL recursively tries to find the stronger dependency relationships for each testing instance based on log likelihood. ${\mathrm{UK}}_{1}\mathrm{DB}$ and ${\mathrm{UK}}_{2}\mathrm{DB}$ had similar time cost for classifying. Although UKDB generally had more training time and classification time than other BNCs, it had higher classification accuracy. Compared to KDB, UKDB delivered markedly lower zero-one loss, also causing too much average computation overhead. The advantage of UTL for improving classification accuracy came at a cost in training time and classification time.

### 4.5. Global Comparison

**Figure 10:**
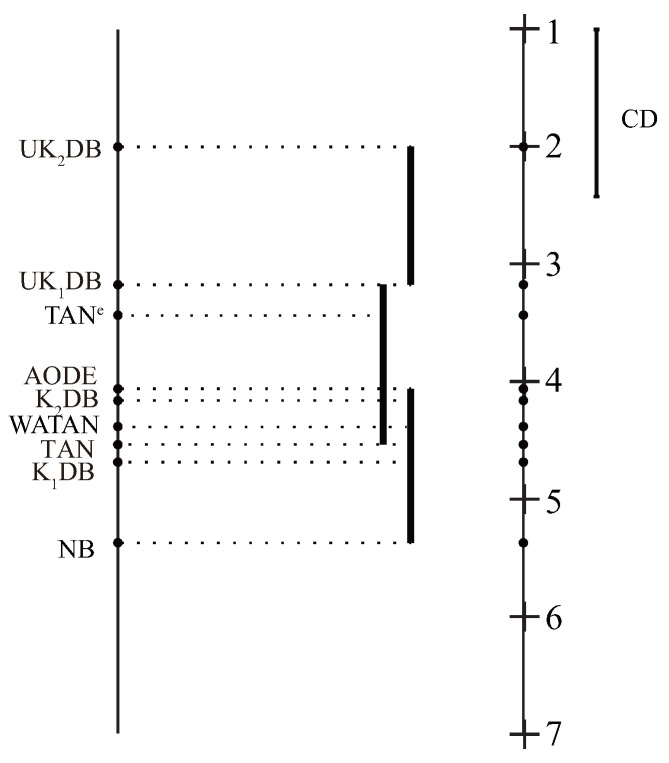
Zero-one loss comparison with the Nemenyi test.

We performed the comparison of our algorithm and other algorithms with the Nemenyi test in Figure 10 proposed by Demšar [53]. If two classifiers’ average ranks are diverse by at least the Critical Difference (CD), their performance differs significantly. The value of CD can be calculated as follows:

(19) $$\mathrm{CD}=q_{\mathrm{ff}}\sqrt{\frac{t(t+1)}{6N}}.$$

where the critical value $q_{\alpha }$ for α = 0.05 and t=9 is 3.102 [53]. Given nine algorithms and 40 datasets, the critical difference (CD) is $\mathrm{CD}=3.102\times \sqrt{9\times (9+1)/(6\times 40)}=1.8996$. We plot the algorithms on the left line according to their average ranks, which are indicated on the parallel right line. Critical Difference (CD) is also presented in the graphs. The lower the position of algorithms, the lower the ranks will be, and hence the better the performance. The algorithms are connected by a line if their differences are not significant. As shown in Figure 10, ${\mathrm{UK}}_{2}\mathrm{DB}$ achieved the lowest mean zero-one loss rank, followed by ${\mathrm{UK}}_{1}\mathrm{DB}$. The average rank of ${\mathrm{UK}}_{2}\mathrm{DB}$ and ${\mathrm{UK}}_{1}\mathrm{DB}$ was significantly better than NB, TAN, $K_{1}\mathrm{DB}$, and $K_{2}\mathrm{DB}$, demonstrating the effectiveness of the proposed universal target learning framework. Compared with the ensemble models AODE, WATAN, and TAN^*e*^, ${\mathrm{UK}}_{2}\mathrm{DB}$ and ${\mathrm{UK}}_{1}\mathrm{DB}$ also achieved lower ranks, but not significantly.

## 5. Conclusions and Future Work

BNCs can graphically represent the dependency relationships implicit in training data and they have been previously demonstrated to be effective and efficient. On the basis of analyzing and summarizing the state-of-the-art BNCs in terms of log likelihood, this paper proposed a novel learning framework for BNC learning, UTL. Our experiments showed its advantages from the comparison results of zero-one loss, RMSE, $F_{1}$-score, etc. UTL can help refine the network structure by fully mining the significant conditional dependencies among attribute values in a specific instance. The application of UTL is time-consuming, and we will seek methods to make it more effective. The research work on extending TL will be very promising. 

## Figures and Tables

**Table 1:** Datasets.

Index	Dataset	Instance	Attribute	Class	Index	Dataset	Instance	Attribute	Class
1	**contact-lenses**	24	4	3	21	**tic-tac-toe**	958	9	2
2	**lung-cancer**	32	56	3	22	**german**	1000	20	2
3	**post-operative**	90	8	3	23	**car**	1728	6	4
4	**zoo**	101	16	7	24	**mfeat-mor**	2000	6	10
5	**echocardiogram**	131	6	2	25	hypothyroid	3163	25	2
6	**lymphography**	148	18	4	26	**kr-vs-kp**	3196	36	2
7	**iris**	150	4	3	27	dis	3772	29	2
8	**teaching-ae**	151	5	3	28	**abalone**	4177	8	3
9	**wine**	178	13	3	29	**waveform-5000**	5000	40	3
10	**autos**	205	25	7	30	**phoneme**	5438	7	50
11	**glass-id**	214	9	3	31	**wall-following**	5456	24	4
12	**hungarian**	294	13	2	32	page-blocks	5473	10	5
13	**heart-disease-c**	303	13	2	33	**thyroid**	9169	29	20
14	**primary-tumor**	339	17	22	34	**sign**	12,546	8	3
15	**horse-colic**	368	21	2	35	**nursery**	12,960	8	5
16	**house-votes-84**	435	16	2	36	**seer_mdl**	18,962	13	2
17	**cylinder-bands**	540	39	2	37	adult	48,842	14	2
18	**balance-scale**	625	4	3	38	**localization**	164,860	5	11
19	**credit-a**	690	15	2	39	**poker-hand**	1,025,010	10	10
20	**pima-ind-diabetes**	768	8	2	40	donation	5,749,132	11	2

**Table 2:** Win/Draw/Loss (W/D/L) of zero-one loss on 40 datasets. AODE, Averaged One-Dependence Estimators; WATAN, Weighted Averaged Tree-Augmented Naive Bayes; UK, *k*-dependence Bayesian classifier with Universal Target Learning (UTL).

W/D/L	NB	TAN	K${}_{1}$DB	K${}_{2}$DB	AODE	WATAN	TAN^*e*^	UK${}_{1}$DB
TAN	20/9/11							
$K_{1}\mathrm{DB}$	22/9/9	9/26/5						
$K_{2}\mathrm{DB}$	19/11/10	17/13/10	15/16/9					
AODE	20/15/5	12/15/13	12/15/13	15/11/14				
WATAN	21/8/11	2/36/2	5/27/8	8/17/15	13/14/13			
TAN^*e*^	21/16/3	26/9/5	17/17/6	13/14/13	10/24/6	16/22/2		
${\mathrm{UK}}_{1}\mathrm{DB}$	24/12/4	18/15/7	21/12/7	16/12/12	15/18/7	19/16/5	13/20/7	
${\mathrm{UK}}_{2}\mathrm{DB}$	26/12/2	26/12/2	28/8/4	28/8/4	24/13/3	24/14/2	18/18/4	16/22/2

**Table 3:** W/D/L of RMSE on 40 datasets.

W/D/L	NB	TAN	K${}_{1}$DB	K${}_{2}$DB	AODE	WATAN	TAN^*e*^	UK${}_{1}$DB
TAN	20/14/6							
$K_{1}\mathrm{DB}$	20/17/3	6/33/1						
$K_{2}\mathrm{DB}$	18/14/8	16/20/4	13/22/5					
AODE	20/18/2	11/21/8	7/23/10	13/15/12				
WATAN	20/16/4	2/38/0	1/35/4	4/22/14	8/24/8			
TAN^*e*^	21/15/4	10/28/2	8/27/5	12/15/13	8/26/6	9/29/2		
${\mathrm{UK}}_{1}\mathrm{DB}$	19/19/2	12/25/3	10/26/4	10/21/9	10/24/6	9/28/3	9/26/5	
${\mathrm{UK}}_{2}\mathrm{DB}$	24/14/2	17/21/2	15/23/2	14/23/2	24/13/3	14/24/2	15/21/4	19/17/4

**Table 4:** W/D/L of the $F_{1}$-score on 40 datasets.

W/D/L	NB	TAN	K${}_{1}$DB	K${}_{2}$DB	AODE	WATAN	TAN^*e*^	UK${}_{1}$DB
TAN	12/22/6							
$K_{1}\mathrm{DB}$	15/19/6	7/31/2						
$K_{2}\mathrm{DB}$	15/16/9	7/29/4	6/31/3					
AODE	15/24/1	7/28/5	7/29/4	10/23/7				
WATAN	13/22/5	2/34/4	7/31/2	7/29/4	7/26/7			
TAN^*e*^	15/20/5	2/32/6	3/29/8	6/26/8	6/28/6	2/33/5		
${\mathrm{UK}}_{1}\mathrm{DB}$	19/17/4	10/27/3	8/30/2	9/26/5	6/30/4	10/27/3	10/30/0	
${\mathrm{UK}}_{2}\mathrm{DB}$	24/14/2	12/25/3	9/26/5	8/26/6	7/28/5	11/27/2	19/17/4	4/33/3

**Table 5:** The H(C)values for the 40 datasets.

Index	Dataset	H(C)	Index	Dataset	H(C)
1	contact-lenses	1.0536	21	tic-tac-toe	0.9281
2	lung-cancer	1.5522	22	german	0.8804
3	post-operative	0.9679	23	car	1.2066
4	zoo	2.3506	24	mfeat-mor	3.3210
5	echocardiogram	0.9076	25	hypothyroid	0.2653
6	lymphography	1.2725	26	kr-vs-kp	0.9981
7	iris	1.5846	27	dis	0.1147
8	teaching-ae	1.5828	28	abalone	1.5816
9	wine	1.5664	29	waveform-5000	1.5850
10	autos	2.2846	30	phoneme	4.7175
11	glass-id	1.5645	31	wall-following	1.7095
12	hungarian	0.9579	32	page-blocks	0.6328
13	heart-disease-c	0.9986	33	thyroid	1.7151
14	primary-tumor	3.7054	34	sign	1.5832
15	horse-colic	0.9533	35	nursery	1.7149
16	house-votes-84	0.9696	36	seer_mdl	0.9475
17	cylinder-bands	0.9888	37	adult	0.7944
18	balance-scale	1.3112	38	localization	2.7105
19	credit-a	0.9911	39	poker-hand	0.9698
20	pima-ind-diabetes	0.9372	40	donation	0.0348

## Appendix A

**Table A1:** Zero-one loss results of NB, TAN, AODE, WATAN, TAN^*e*^, $K_{1}\mathrm{DB}$, $K_{2}\mathrm{DB}$, ${\mathrm{UK}}_{1}\mathrm{DB}$, and ${\mathrm{UK}}_{2}\mathrm{DB}$.

Index	Datasets	NB	TAN	AODE	WATAN	TAN^*e*^	K${}_{1}$DB	K${}_{2}$DB	UK${}_{1}$DB	UK${}_{2}$DB
1	**contact-lenses**	0.3750	0.3750	0.3750	0.4583	0.3750	0.2917	0.2500	0.3333	0.3333
2	**lung-cancer**	0.4375	0.5938	0.5000	0.6250	0.6563	0.5938	0.5625	0.5625	0.5938
3	**post-operative**	0.3444	0.3667	0.3333	0.3667	0.3444	0.3444	0.3778	0.3556	0.3333
4	**zoo**	0.0297	0.0099	0.0297	0.0198	0.0099	0.0495	0.0495	0.0198	0.0297
5	**echocardiogram**	0.3359	0.3282	0.3206	0.3282	0.3282	0.3053	0.3435	0.3206	0.3282
6	**lymphography**	0.1486	0.1757	0.1689	0.1689	0.1689	0.1757	0.2365	0.1486	0.1554
7	**iris**	0.0867	0.0800	0.0867	0.0800	0.0867	0.0867	0.0867	0.0867	0.0733
8	**teaching-ae**	0.4967	0.5497	0.4901	0.5364	0.5166	0.5430	0.5364	0.4702	0.4503
9	**wine**	0.0169	0.0337	0.0225	0.0337	0.0337	0.0393	0.0225	0.0225	0.0169
10	**autos**	0.3122	0.2146	0.2049	0.2146	0.2000	0.2146	0.2049	0.2049	0.2000
11	**glass-id**	0.2617	0.2196	0.2523	0.2196	0.2103	0.2243	0.2196	0.2150	0.2056
12	**hungarian**	0.1599	0.1701	0.1667	0.1735	0.1599	0.1701	0.1803	0.1633	0.1463
13	**heart-disease-c**	0.1815	0.2079	0.2013	0.2046	0.1881	0.2079	0.2244	0.1914	0.2013
14	**primary-tumor**	0.5457	0.5428	0.5752	0.5428	0.5575	0.5693	0.5723	0.5457	0.5339
15	**horse-colic**	0.2174	0.2092	0.2011	0.2120	0.2038	0.2174	0.2446	0.2065	0.2092
16	**house-votes-84**	0.0943	0.0552	0.0529	0.0529	0.0552	0.0690	0.0506	0.0552	0.0391
17	**cylinder-bands**	0.2148	0.2833	0.1889	0.2463	0.1833	0.2278	0.2259	0.1815	0.1815
18	**balance-scale**	0.2720	0.2736	0.2832	0.2736	0.2784	0.2816	0.2784	0.2640	0.2640
19	**credit-a**	0.1406	0.1507	0.1391	0.1507	0.1391	0.1551	0.1464	0.1348	0.1377
20	**pima-ind-diabetes**	0.2448	0.2383	0.2383	0.2370	0.2383	0.2422	0.2448	0.2357	0.2331
21	**tic-tac-toe**	0.3069	0.2286	0.2651	0.2265	0.2724	0.2463	0.2035	0.2317	0.1733
22	**german**	0.2530	0.2730	0.2480	0.2760	0.2590	0.2760	0.2890	0.2560	0.2680
23	**car**	0.1400	0.0567	0.0816	0.0567	0.0579	0.0567	0.0382	0.0741	0.0723
24	**mfeat-mor**	0.3140	0.2970	0.3145	0.2980	0.3050	0.2990	0.3060	0.3080	0.3070
25	hypothyroid	0.0149	0.0104	0.0136	0.0104	0.0092	0.0107	0.0107	0.0098	0.0101
26	**kr-vs-kp**	0.1214	0.0776	0.0842	0.0776	0.0566	0.0544	0.0416	0.0485	0.0454
27	dis	0.0159	0.0159	0.0130	0.0154	0.0162	0.0146	0.0138	0.0141	0.0127
28	**abalone**	0.4762	0.4587	0.4472	0.4582	0.4554	0.4633	0.4563	0.4539	0.4554
29	**waveform-5000**	0.2006	0.1844	0.1462	0.1844	0.1650	0.1820	0.2000	0.1598	0.1642
30	**phoneme**	0.2615	0.2733	0.2392	0.2345	0.2429	0.2120	0.1984	0.1901	0.1841
31	**wall-following**	0.1054	0.0554	0.0370	0.0550	0.0462	0.0462	0.0401	0.0389	0.0295
32	page-blocks	0.0619	0.0415	0.0338	0.0418	0.0342	0.0433	0.0391	0.0364	0.0358
33	**thyroid**	0.1111	0.0720	0.0701	0.0723	0.0726	0.0693	0.0706	0.0835	0.0669
34	**sign**	0.3586	0.2755	0.2821	0.2752	0.2713	0.2881	0.2539	0.2713	0.2572
35	**nursery**	0.0973	0.0654	0.0730	0.0654	0.0617	0.0654	0.0289	0.0702	0.0555
36	**seer_mdl**	0.2379	0.2376	0.2328	0.2374	0.2332	0.2367	0.2555	0.2363	0.2367
37	adult	0.1592	0.1380	0.1493	0.1380	0.1326	0.1385	0.1383	0.1382	0.1347
38	**localization**	0.4955	0.3575	0.3596	0.3575	0.3610	0.3706	0.2964	0.3319	0.3112
39	**poker-hand**	0.4988	0.3295	0.4812	0.3295	0.0763	0.3291	0.1961	0.0618	0.0752
40	donation	0.0002	0.0000	0.0002	0.0000	0.0000	0.0000	0.0000	0.0001	0.0000

**Table A2:** RMSE results of NB, TAN, AODE, WATAN, TAN^*e*^, $K_{1}\mathrm{DB}$, $K_{2}\mathrm{DB}$, ${\mathrm{UK}}_{1}\mathrm{DB}$, and ${\mathrm{UK}}_{2}\mathrm{DB}$.

Index	Datasets	NB	TAN	AODE	WATAN	TAN^*e*^	K${}_{1}$DB	K${}_{2}$DB	UK${}_{1}$DB	UK${}_{2}$DB
1	**contact-lenses**	0.5017	0.6077	0.5258	0.5737	0.5736	0.5024	0.4996	0.5136	0.5033
2	**lung-cancer**	0.6431	0.7623	0.6915	0.7662	0.7069	0.7523	0.7313	0.6942	0.7391
3	**post-operative**	0.5103	0.5340	0.5215	0.5358	0.5157	0.5289	0.5632	0.5256	0.5289
4	**zoo**	0.1623	0.1309	0.1536	0.1341	0.1313	0.1984	0.1815	0.1426	0.1428
5	**echocardiogram**	0.4896	0.4886	0.4903	0.4890	0.4852	0.4846	0.4889	0.4878	0.4891
6	**lymphography**	0.3465	0.3813	0.3556	0.3857	0.3761	0.3726	0.4362	0.3614	0.3571
7	**iris**	0.2545	0.2441	0.2544	0.2435	0.2505	0.2435	0.2447	0.2628	0.2407
8	**teaching-ae**	0.6204	0.6300	0.6117	0.6242	0.6191	0.6332	0.6286	0.6286	0.6262
9	**wine**	0.1134	0.1746	0.1245	0.1748	0.1583	0.1761	0.1501	0.1355	0.1374
10	**autos**	0.5190	0.4475	0.4397	0.4420	0.4362	0.4460	0.4399	0.4252	0.4385
11	**glass-id**	0.4353	0.4109	0.4235	0.4087	0.4036	0.4223	0.4205	0.4179	0.4020
12	**hungarian**	0.3667	0.3429	0.3476	0.3418	0.3315	0.3380	0.3552	0.3534	0.3444
13	**heart-disease-c**	0.3743	0.3775	0.3659	0.3783	0.3583	0.3810	0.3963	0.3802	0.3877
14	**primary-tumor**	0.7084	0.7170	0.7155	0.7166	0.7154	0.7190	0.7262	0.7085	0.7048
15	**horse-colic**	0.4209	0.4205	0.4015	0.4215	0.3951	0.4131	0.4348	0.4164	0.4247
16	**house-votes-84**	0.2997	0.2181	0.1994	0.2181	0.2126	0.2235	0.1969	0.2221	0.1779
17	**cylinder-bands**	0.4291	0.4358	0.4080	0.4277	0.3973	0.4435	0.4431	0.4077	0.4083
18	**balance-scale**	0.4431	0.4344	0.4350	0.4344	0.4414	0.4384	0.4323	0.4279	0.4286
19	**credit-a**	0.3350	0.3415	0.3271	0.3407	0.3300	0.3416	0.3480	0.3336	0.3355
20	**pima-ind-diabetes**	0.4147	0.4059	0.4078	0.4059	0.4044	0.4054	0.4074	0.4095	0.4082
21	**tic-tac-toe**	0.4309	0.4023	0.3995	0.4023	0.4216	0.4050	0.3772	0.4134	0.3421
22	**german**	0.4204	0.4367	0.4161	0.4373	0.4206	0.4389	0.4665	0.4364	0.4531
23	**car**	0.3395	0.2405	0.3022	0.2406	0.2565	0.2404	0.2031	0.2426	0.2358
24	**mfeat-mor**	0.4817	0.4657	0.4710	0.4660	0.4686	0.4665	0.4707	0.4673	0.4652
25	hypothyroid	0.1138	0.0955	0.1036	0.0951	0.0933	0.0956	0.0937	0.0931	0.0928
26	**kr-vs-kp**	0.3022	0.2358	0.2638	0.2358	0.2417	0.2159	0.1869	0.1992	0.1866
27	dis	0.1177	0.1103	0.1080	0.1098	0.1084	0.1072	0.1024	0.1059	0.1021
28	**abalone**	0.5871	0.5638	0.5559	0.5637	0.5596	0.5653	0.5646	0.5654	0.5625
29	**waveform-5000**	0.4101	0.3611	0.3257	0.3610	0.3417	0.3618	0.3868	0.3474	0.3568
30	**phoneme**	0.4792	0.5048	0.4689	0.4676	0.4796	0.4385	0.4195	0.4055	0.4091
31	**wall-following**	0.3083	0.2245	0.1829	0.2223	0.1989	0.2050	0.1930	0.1884	0.1642
32	page-blocks	0.2331	0.1894	0.1629	0.1895	0.1646	0.1940	0.1811	0.1739	0.1696
33	**thyroid**	0.3143	0.2443	0.2425	0.2431	0.2403	0.2414	0.2423	0.2493	0.2331
34	**sign**	0.5270	0.4615	0.4702	0.4614	0.4682	0.4759	0.4370	0.4581	0.4387
35	**nursery**	0.2820	0.2194	0.2503	0.2194	0.2252	0.2193	0.1776	0.2177	0.2003
36	**seer_mdl**	0.4233	0.4131	0.4112	0.4132	0.4071	0.4131	0.4340	0.4214	0.4219
37	adult	0.3409	0.3076	0.3245	0.3076	0.3024	0.3071	0.3089	0.3167	0.3132
38	**localization**	0.6776	0.5656	0.5856	0.5656	0.5776	0.5767	0.5106	0.5471	0.5169
39	**poker-hand**	0.5801	0.4987	0.5392	0.4987	0.4390	0.4987	0.4055	0.3736	0.3500
40	donation	0.0123	0.0050	0.0114	0.0050	0.0079	0.0050	0.0046	0.0064	0.0055

**Table A3:** $F_{1}$-score results of NB, TAN, AODE, WATAN, TAN^*e*^, $K_{1}\mathrm{DB}$, $K_{2}\mathrm{DB}$, ${\mathrm{UK}}_{1}\mathrm{DB}$, and ${\mathrm{UK}}_{2}\mathrm{DB}$.

Index	Datasets	NB	TAN	AODE	WATAN	TAN^*e*^	K${}_{1}$DB	K${}_{2}$DB	UK${}_{1}$DB	UK${}_{2}$DB
1	**contact-lenses**	0.4540	0.3778	0.3856	0.3377	0.3619	0.5748	0.6875	0.4878	0.4878
2	**lung-cancer**	0.5699	0.4211	0.5030	0.3922	0.3545	0.4163	0.4359	0.4188	0.3799
3	**post-operative**	0.2658	0.2981	0.3065	0.2981	0.3025	0.3185	0.3068	0.3154	0.2898
4	**zoo**	0.9237	0.9948	0.9296	0.9756	0.9824	0.8879	0.8805	0.9622	0.9364
5	**echocardiogram**	0.5631	0.5406	0.5658	0.5406	0.5406	0.5774	0.5078	0.5563	0.5507
6	**lymphography**	0.8720	0.7221	0.6856	0.5614	0.5618	0.7896	0.5281	0.8054	0.6357
7	**iris**	0.9133	0.9200	0.9133	0.9200	0.9133	0.9133	0.9133	0.9133	0.9267
8	**teaching-ae**	0.5011	0.4515	0.5051	0.4649	0.4832	0.4588	0.4660	0.5263	0.5481
9	**wine**	0.9832	0.9664	0.9780	0.9664	0.9664	0.9606	0.9780	0.9773	0.9832
10	**autos**	0.7825	0.8457	0.5792	0.8482	0.8606	0.8482	0.8596	0.8691	0.8685
11	**glass-id**	0.7400	0.7863	0.7564	0.7863	0.7968	0.7807	0.7864	0.7880	0.7994
12	**hungarian**	0.8224	0.8115	0.8148	0.8082	0.8232	0.8132	0.8033	0.8215	0.8390
13	**heart-disease-c**	0.8169	0.7894	0.7972	0.7926	0.8095	0.7897	0.7738	0.8070	0.7961
14	**primary-tumor**	0.3185	0.3307	0.2924	0.3139	0.2894	0.2848	0.2891	0.2880	0.2949
15	**horse-colic**	0.7701	0.7730	0.7849	0.7704	0.7781	0.7645	0.7391	0.7784	0.7706
16	**house-votes-84**	0.9021	0.9419	0.9444	0.9444	0.9419	0.9276	0.9468	0.9422	0.9591
17	**cylinder-bands**	0.7628	0.6799	0.7979	0.7310	0.8041	0.7570	0.7588	0.8084	0.8088
18	**balance-scale**	0.5051	0.5041	0.4974	0.5041	0.5007	0.4985	0.5020	0.5108	0.5107
19	**credit-a**	0.8565	0.8469	0.8586	0.8470	0.8591	0.8424	0.8515	0.8639	0.8607
20	**pima-ind-diabetes**	0.7287	0.7317	0.7327	0.7334	0.7290	0.7311	0.7272	0.7319	0.7365
21	**tic-tac-toe**	0.6358	0.7300	0.6847	0.7330	0.6283	0.7131	0.7649	0.6853	0.7825
22	**german**	0.6880	0.6647	0.6824	0.6599	0.6569	0.6507	0.6451	0.6578	0.6509
23	**car**	0.6607	0.9175	0.7569	0.9175	0.8903	0.9175	0.9354	0.8685	0.8464
24	**mfeat-mor**	0.6759	0.7001	0.6797	0.6994	0.6927	0.6988	0.6905	0.6863	0.6874
25	hypothyroid	0.9251	0.9424	0.9299	0.9424	0.9488	0.9409	0.9405	0.9469	0.9447
26	**kr-vs-kp**	0.8782	0.9223	0.9154	0.9223	0.9432	0.9455	0.9583	0.9514	0.9546
27	dis	0.7460	0.5674	0.7799	0.5818	0.4959	0.6196	0.6870	0.6610	0.7041
28	**abalone**	0.5047	0.5367	0.5476	0.5372	0.5338	0.5334	0.5384	0.5396	0.5400
29	**waveform-5000**	0.7886	0.8159	0.8532	0.8159	0.8351	0.8182	0.8002	0.8395	0.8350
30	**phoneme**	0.6971	0.6778	0.6551	0.7235	0.7087	0.7838	0.7908	0.7823	0.7781
31	**wall-following**	0.8742	0.9333	0.9564	0.9337	0.9445	0.9440	0.9514	0.9526	0.9613
32	page-blocks	0.7530	0.8219	0.8324	0.8199	0.8300	0.8130	0.8174	0.8189	0.8317
33	**thyroid**	0.6103	0.6065	0.6897	0.5947	0.6108	0.6114	0.5752	0.6503	0.6426
34	**sign**	0.6373	0.7228	0.7163	0.7230	0.7275	0.7099	0.7456	0.7269	0.7412
35	**nursery**	0.5709	0.6130	0.6047	0.6131	0.6134	0.6131	0.7053	0.6138	0.6509
36	**seer_mdl**	0.7363	0.7283	0.7364	0.7285	0.7303	0.7284	0.7082	0.7316	0.7292
37	adult	0.7986	0.8063	0.8070	0.8063	0.8092	0.8052	0.8028	0.8112	0.8133
38	**localization**	0.2396	0.3955	0.3686	0.3953	0.3707	0.3817	0.4742	0.4060	0.4383
39	**poker-hand**	0.0668	0.1919	0.0789	0.1920	0.1920	0.1912	0.2552	0.2749	0.2826
40	donation	0.9872	0.9977	0.9878	0.9977	0.9973	0.9977	0.9983	0.9947	0.9975
